# Simulation of Single-Event Transient Effect for GaN High-Electron-Mobility Transistor

**DOI:** 10.3390/mi14101948

**Published:** 2023-10-19

**Authors:** Zhiheng Wang, Yanrong Cao, Xinxiang Zhang, Chuan Chen, Linshan Wu, Maodan Ma, Hanghang Lv, Ling Lv, Xuefeng Zheng, Wenchao Tian, Xiaohua Ma, Yue Hao

**Affiliations:** 1 School of Electronics & Mechanical Engineering, Xidian University, Xi’an 710071, China; 21041211891@stu.xidian.edu.cn (Z.W.); 22041212821@stu.xidian.edu.cn (X.Z.); 22041212707@stu.xidian.edu.cn (C.C.); 22043222985@stu.xidian.edu.cn (L.W.); 20041211840@stu.xidian.edu.cn (M.M.);; 2 State Key Discipline Laboratory of Wide Bandgap Semiconductor Technology, School of Microelectronics, Xidian University, Xi’an 710071, Chinayhao@xidian.edu.cn (Y.H.)

**Keywords:** GaN HEMTs, single-event transient effects, charge collection

## Abstract

A GaN high-electron-mobility transistor (HEMT) was simulated using the semiconductor simulation software Silvaco TCAD in this paper. By constructing a two-dimensional structure of GaN HEMT, combined with key models such as carrier mobility, the effects of a different state, different incidence position, different drain voltage, different LET values, and a different incidence angle on the single-event transient effect of GaN HEMT are simulated. LET stands for the linear energy transfer capacity of a particle, which refers to the amount of energy transferred by the particle to the irradiated substance on the unit path. The simulation results show that for GaN HEMTs, the single-event transient effect is more obvious when the device is in off-state than in on-state. The most sensitive location of GaN HEMTs to the single-event effect is in the region near the drain. The peak transient current increases with the increase in the drain bias and incident ion LET values. The drain charge collection time increases with the angle of incidence of heavy ion.

## 1. Introduction

At present, silicon-based devices are still the most widely used devices in the space field, and research on its single-event effect is becoming more and more perfect, but the physical characteristics of silicon materials themselves greatly limit the further development and application of silicon-based devices in the field of radiation resistance, so GaN, as a representative material of a new generation of semiconductor materials, has gradually attracted people’s attention. At present, according to the type of gate, GaN HEMTs can be divided into Schottky gate HEMTs and MIS-HEMTs; for MIS-HEMTs, an insulating medium is introduced under the gate, which well suppresses the gate leakage current, but due to the introduction of an insulating medium, the distance from the gate metal to the two-dimensional electron gas channel increases, which weakens the gate control ability of the device [1]. Depending on whether the threshold voltage is greater than 0, GaN HEMTs can be divided into depletion-mode HEMTs and enhancement-mode HEMTs. Mainstream enhancement-mode HEMTs include recessed-gate HEMTs and P-GaN HEMTs. Recessed-gate HEMTs suppress the polarization effect by thinning the thickness of the AlGaN layer so that the threshold voltage is greater than 0, but the weakening of the polarization effect weakens the current output capability of the device and significantly reduces the maximum drain current. P-GaN HEMTs adjust the energy band of the gate region by adding a P-doped GaN layer below the gate, thereby depleting the two-dimensional electron gas and making the threshold voltage greater than 0. Although this practice did not affect the current output capability, the addition of a GaN layer below the gate significantly increased the distance from the gate metal to the 2-DEG channel, and the gate control capability of the device was significantly reduced [2]. Single-event transient effects refer to a phenomenon in which a single high-energy particle is incident to a semiconductor device. Due to ionization energy deposition, a large number of electron–hole pairs are induced in the device, and a pulse current is formed after the electron–hole pair is collected by the electric field, resulting in a change in the working state of the device. Compared with silicon-based devices, GaN HEMTs have better physical characteristics, such as large electron mobility, high electron density, large band gap, high breakdown field strength, high temperature resistance, and high electric pressure ability [3,4,5,6]. Some studies have shown that GaN HEMTs have a strong resistance to total dose effect due to a large band gap [7,8,9], but GaN HEMTs are more sensitive to single-event effects. Total dose effect is that a large number of radiation particles enter the semiconductor device material and ionization occurs with the electrons outside the nucleus of the material to produce additional charges, which accumulate in the device and induce interface states at the interface, resulting in the gradual degradation of device performance and even the eventual loss. In space, due to the different particle types, energies, angle of incidence, and the state of the device when the particle is incident, the effect of the single-event effects on the device is complex [10]. For GaN HEMTs, many single-event effect problems have not been solved. Due to different theories on the charge collection mechanism, the radiation effect of devices under various conditions is not comprehensive [11]. Samsel et al. used AlGaN/GaN MOS-HEMT devices for heavy ion radiation and found that the devices exhibited significant charge collection at the gate [12]. Onoda et al. conducted off-state radiation experiments on AlGaN/GaN HEMTs to study the effect of displacement damage on the enhanced charge collection ability and found that the back-channel effect has a significant contribution to the enhanced charge collection mechanism in the single-event transient effect compared with the parasitic transistor effect [13]. Khachatrian et al. studied the single-event transient effects of AlGaN/GaN HEMTs by carrier injection through single-photon absorption by ultraviolet pulses, and the results showed that the transient current peak and shape largely depend on the position of the injected carriers and change with the gate and drain bias conditions [14]. Hu et al. simulated heavy ion radiation at different incidence angles through software simulation and found that the most serious incidence of the device was not from the vertical incidence at the end of the field plate but from the low angle incidence in the middle of the field plate [15]. However, the single-event transient effects are not fully studied under different device bias states and different incident particle conditions, and it is important to conduct a comprehensive study for practical applications. Therefore, in this paper, Silvaco TCAD software [16] is used to study the drain transient current of GaN HEMTs under different gate voltages, drain voltages, heavy ion LET values, incidence positions, incidence angles, etc., combined with the charge collection mechanism to analyze the influence of these factors on the single-event transient effects and use the simulation results to illustrate the sensitive region of GaN HEMTs for the single-event effects, which provides a theoretical basis for optimizing the radiation resistance of the device.

## 2. Simulation Model Structure

### 2.1. Device Model

**Figure 1:**
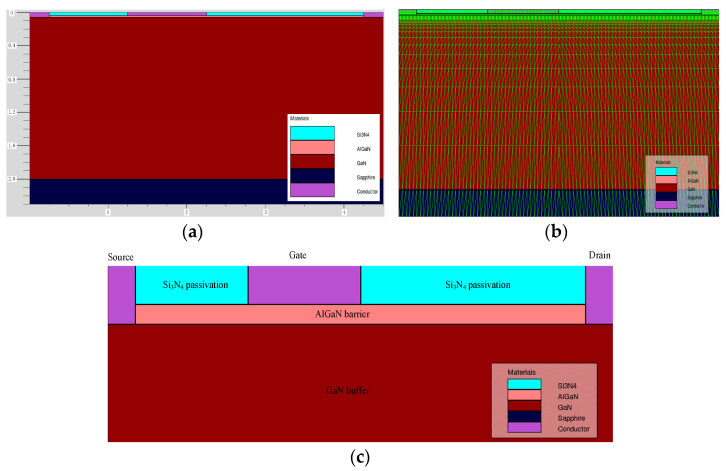
(**a**) Two-dimensional structure simulation diagram. (**b**) Schematic diagram of meshing. (**c**) Enlarged view of the heterojunction part of GaN HEMT.

The simulation work in this article was carried out using Silvaco TCAD (Technology Computer Aided Design) simulation software. Based on the GaN HEMTs provided by Xidian University, the device model was built using Silvaco TCAD (Version 5.0.10.R, Santa Clara, CA 95054, USA). The generated two-dimensional structure diagram, schematic diagram of meshing, and the enlarged view of the heterojunction part of the device are shown in Figure 1a, Figure 1b, and Figure 1c, respectively.

The material and meshing of each part of the simulated device and the device structure parameters during the simulation process are listed in Table 1. The structural parameters are based on the actual device. In order to ensure the consistency of simulation and experiment, the source electrode and drain electrode in the simulation structure are etched into the GaN buffer layer so that the electrodes can be directly in contact with the 2DEG (two-dimensional electron gas channel) [17]. The simulation results show that the device characteristics are close to the actual device, which ensures the reliability of the simulation results.

**Figure 2:**
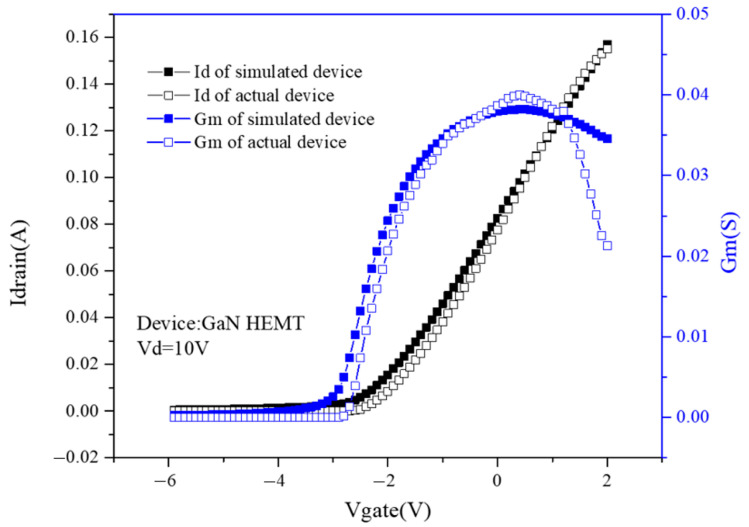
Transfer curve and transconductance curve of the simulated device and the actual device.

The gate contact work function is 5.23. The setting of GaN material parameters such as band gap energy and relative permittivity in Table 2 are based on [18,19] settings. In addition, the source and drain of the device are ohmic contact, and the gate is Schottky contact. The device transfer characteristic curve and transconductance curve are obtained as shown in Figure 2.

In order to make the device characteristics in the simulation closer to the actual device characteristics, the electrode work function, polar scale parameter, and background carrier concentration are adjusted during the simulation. As can be seen from Figure 2, after the device is turned on, the current is 0.16 A when the voltage of the gate is 2 V, and the maximum transconductance is 0.038 S. The device characteristics are consistent with the actual device.

### 2.2. The Physical Model Used in the Simulation

Considering the possible recombination during carrier transport, the SRH composite model and the Auger composite model are introduced. In order to simulate the change in carrier mobility in the simulation, the carrier mobility model Conmob, the low-field mobility model Albrct.n, and the high-field mobility model Gansat are also introduced [20,21]. Particle transfers energy to the device material through collision ionization, so the collision ionization model is added. Considering that when ion is incident, a large number of electron–hole pairs are generated quickly in GaN HEMT, the Fermi–Dirac carrier statistical model is introduced because it is more accurate for the calculation of carriers at high density.

**Figure 3:**
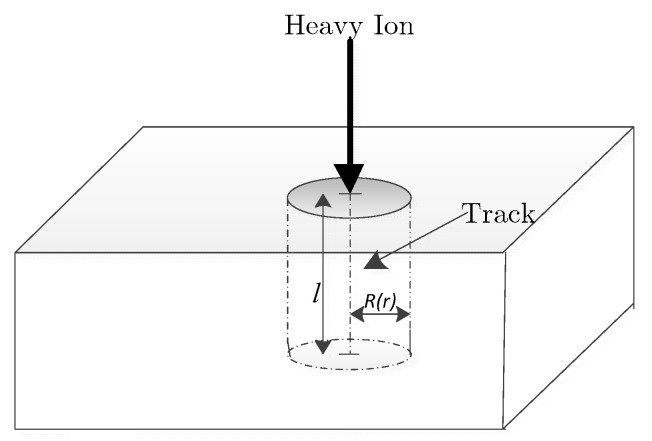
Heavy ion bombardment model.

The particle incidence model uses a heavy ion bombardment model [22,23], and the schematic diagram is shown in Figure 3.

When heavy ion is incident in the sensitive region of the device, due to ionization, lots of electron–hole pairs will be formed near the incidence trajectory, in which the electrons are quickly collected by the high-voltage region, and the holes are collected by the low-voltage region, thus forming a large pulse current, which affects the state of the device. The number of carriers produced by heavy ion incidence *G (l, r, t)* can be expressed below.

(1) $$G(l,r,t)=G_{LET}(l)R(r)T(t)$$

*G_LET_* (*l*) is the linear energy transfer; it is defined as the energy lost by a particle on the entire path of the bombarded semiconductor material, the magnitude of which is determined by the type, energy, and range of the particle. It is the integral of *LET* on the path. The formula of *LET* is:

(2) $$LET=\frac{1}{\rho }\frac{dE}{dx}$$

where *ρ* represents the density of the incident substance, and *dE*/*dx* is the transfer of energy per unit path length.

The spatial distribution of *R*(*r*) uses the Gaussian distribution and can be expressed as:

(3) $$R(r)=\exp{\left(-\frac{r}{radius}\right)}^{2}$$

where *r* is the radial distance from the center of the orbit to the point, and *radius* is the radius of the incident particle beam. The time dependence of charge generation *T*_(*t*)_ is controlled by the *T_C_* parameter through two functions. If *T_C_* is 0, *T*_(*t*)_ = deltafunction(*t* − *T_0_*); if *T_C_* is greater than 0, then:

(4) $$T(t)=\frac{2e-{\left(\frac{t-T_{0}}{T_{C}}\right)}^{2}}{T_{C}\sqrt{\pi }erfc(\frac{-T_{0}}{T_{C}})}$$

where *T*_0_ refers to the peak time when the particle generates a pulse. *T_C_* is Gaussian function time width of the particle-generated pulse.

## 3. The Simulation Results and Analysis of Single-Event Transient Effects of GaN HEMT

**Figure 4:**
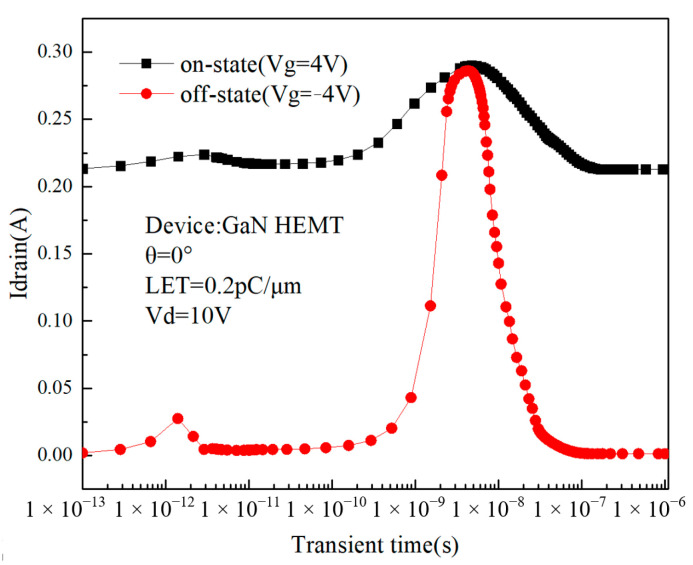
Drain current curves of the device in on- and off-states.

The gate voltage controls the opening and closing of the 2DEG channel of the device, and the status of the device has a great influence on the single-event transient effects. By controlling the gate voltage, the single-event transient effects in different gate bias states of the device are simulated, respectively. Simulations are performed in two different states: on (Vg = 4 V) and off (Vg = −4 V). The incidence trajectory radius is 0.05 μm. Figure 4 shows the single-event transient current of the device in the on and off states, respectively. In off-state, the drain current of the device is 0.0020 A before the particle incident and 0.286 A when the single-event transient effect occurs; in on-state, the drain current of the device is 0.2133 A before the particle incident and 0.2901 A after the single-event transient effect. In off-state, the peak drain transient current is approximately 146.71 times the initial state, while in on-state, the peak drain transient current is only 1.36 times the initial current. It can be concluded that for GaN HEMTs, the single-event effect is more severe in off-state than in on-state. In 2021, Sehra et al. used GaN HEMT devices to conduct single-particle transient experiments at different gate voltages [10], and the simulation results in this paper were consistent with the results of Sehra et al. In Figure 4, transient time refers to the time from when the particle is incident into the device to when the electron–hole pairs are fully collected and the transient current peak ends. Since this period of time is extremely short, it is called transient time.

**Figure 5:**
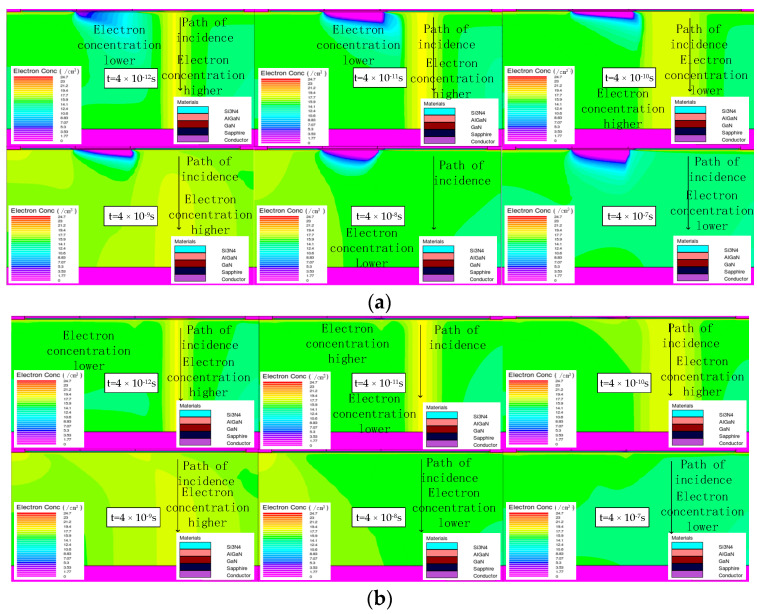
(**a**) The internal electron concentration of the device at different times in off-state. (**b**) The internal electronic concentration of the device at different times in on-state.

Figure 5a,b show the distribution of the electron concentration at different times when the device is in off-state and on-state, respectively. It can be seen from Figure 5a that after the incident of heavy ion, many electrons are excited on its incidence path, and these electrons are then collected by the electric field, appearing as drain current pulses. Within 4 × 10^−9^ s from the moment of heavy ion incidence, the area of the region with a higher electron concentration inside the device increases, and the charge collection rate and recombination rate are less than the charge diffusion rate during this time. Between 4 × 10^−9^ s and 4 × 10^−7^ s, the area of the region with higher electron concentration decreases significantly, indicating that the charge collection rate and recombination rate are greater than the charge diffusion rate during this time. The current pulses in Figure 4 also appear during these time periods, coinciding with the phenomenon in the electron concentration distribution image.

It can be seen from Figure 5a that when the device is in off-state (Vg = −4 V), the electron concentration in the region below the gate is very low, and there is no significant increase in the electron concentration in this region until the charge is collected. This indicates that due to the negative bias of the gate, the 2DEG channel is turned off, and the drain cannot collect charge through the channel, so it can be speculated that the charge is collected by the drain through the charge leakage path under the 2DEG channel, which has a more serious impact on the device. When the device is in on-state (Vg = 4 V), as shown in Figure 5b, the 2DEG channel is turned on, so the ionized charge can be directly collected by the drain through the channel without seriously affecting the device.

**Figure 6:**
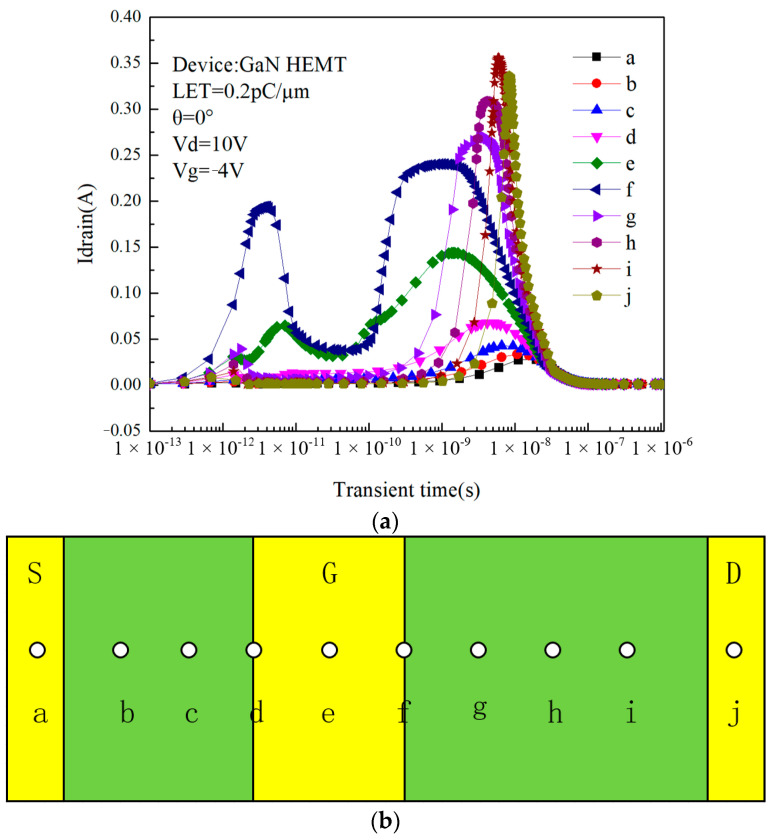
(**a**) Relationship between drain current and the incidence location over time. (**b**) Schematic diagram of the incidence location.

In fact, as can be seen in Figure 4, there are two peaks in the drain current. In order to investigate the cause of these two current peaks, the incidence position of heavy ion was changed, and the peak of the drain current was observed. Figure 6a shows the drain pulse current I_drain_ when different incident locations are selected during simulation. The incidence location is selected between the source and drain in the direction of the 2DEG length. Ten points, A–J, are selected, and the selected positions are shown in Figure 6b. Combined with the previous analysis, considering the worst scenario, heavy ion is incident when the device is in off-state.

**Figure 7:**
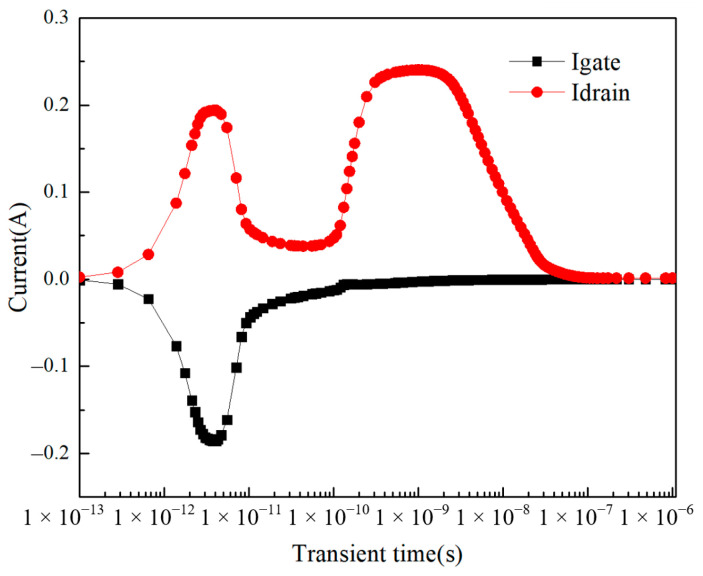
When the heavy ion is incident at point f, there is a relationship between gate current and drain current over time.

As can be seen from the figure, the effect of changing the incidence position of the heavy ion on the transient current is significant. For the first peak that occurs earlier, the transient current is greatest at point f, with a peak of 0.194 A, while for the second peak that occurs later, the transient current is greatest at point i, with a peak of 0.336 A. The reason for the occurrence of two current peaks can be explained as follows: due to the different locations where the charge is collected by the device, the peak current occurs at different times. When heavy ion is incident inside the device, the ionized charge is collected by the device in two parts. For the first peak that occurs earlier, the charge collection sites are the gate and drain. After the moment of heavy ion incidence, the ionized electron–hole pairs are close to the device surface and closer to the electrodes so that they can be rapidly collected by the gate and drain. Figure 7 shows the relationship between gate current and drain current with time when heavy ion is incident at point f. It can be seen from the figure that at the moment of the appearance of the first peak (between 1 × 10^−12^ s and 1 × 10^−11^ s), the gate current and drain current are equal in magnitude and electrically opposite. It is proved that there are equal but electrically opposite charges that are collected by the gate and drain at the same time.

**Figure 8:**
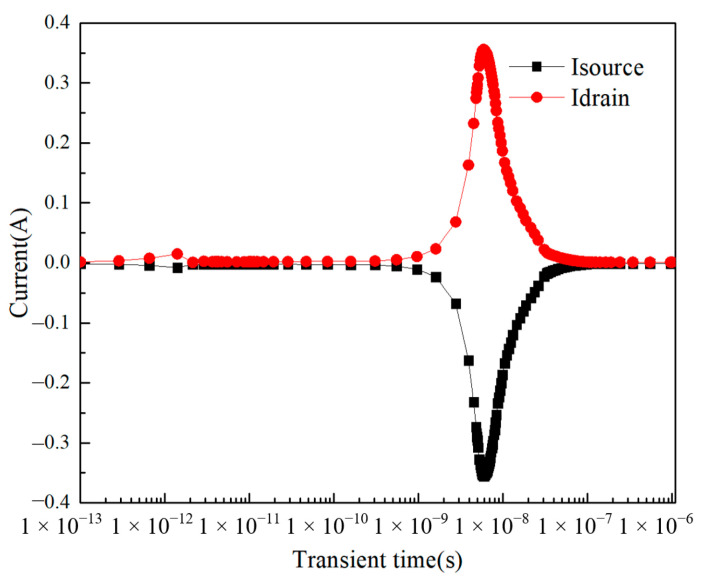
When the heavy ion is incident at point i, there is a relationship between gate current and drain current over time.

For the second peak, which occurs later, charge is collected at the drain and source. The heavy ion incidents then ionize the electron–hole pairs inside the device, where the charge takes longer to move because of the greater distance from the source and drain, resulting in the peak that occurs later than the first peak current. Under the action of the source–drain electric field, the charge is collected by the source and drain, forming the second current peak. The figure shows the relationship between the source current and drain current when the heavy ion is incident at point i. As can be seen from Figure 8, the above inference is confirmed by the fact that at the moment of the occurrence of the second peak (between 1 × 10^−9^ s and 1 × 10^−7^ s), the source has a current equal to the size of the drain and the opposite charge, which proves the inference above.

It is important to note that the source current and drain current in Figure 8 are not mirrored. The source current and drain current are not completely mirrored; at the second peak current (between 1 × 10^−9^ s–1 × 10^−7^ s), the source current and drain current are equal in magnitude and electrically opposite; but at the first peak current, the current magnitude of the two electrodes is not equal.

**Figure 9:**
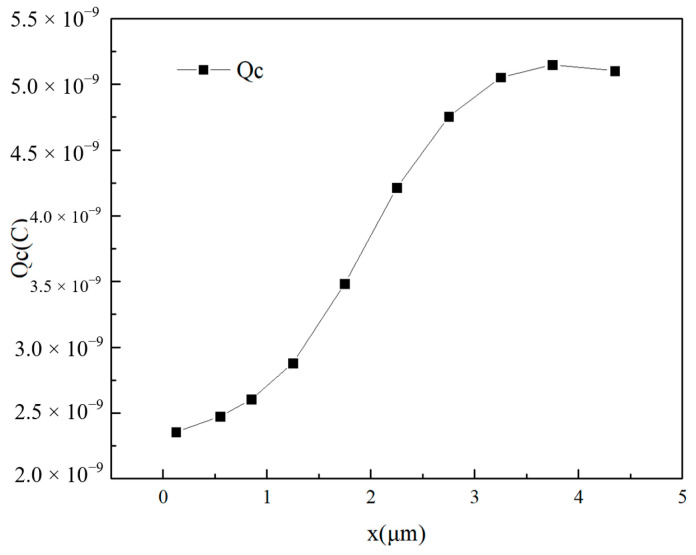
The total charge Qc (the integration of the current over time, which is the area enclosed by the image and the x-axis) collected by the drain changes with the incidence position of the heavy ion.

Figure 9 shows the total charge collected by the drain as a function of the heavy ion incidence position. x represents the distance from the source of the point of incidence. It can be seen from the figure that during the change in the incidence position from source to drain, the amount of charge collected first increases and then decreases slightly, and maximum values appear near the edge of the drain. It can be inferred that for GaN HEMTs, the drain is the most sensitive region for single-event transient effects.

**Figure 10:**
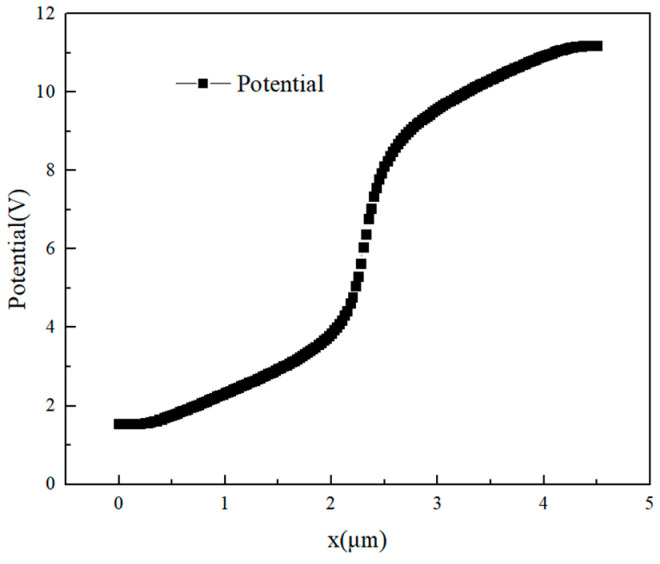
The internal potential of the device changes with the position.

For GaN HEMTs, when heavy ion enters the device, a large number of electron–hole pairs are ionized, which are collected under the action of the gate–drain electric field or source–drain electric field. Figure 10 shows the relationship between the internal potential of the GaN HEMT and the position. It can be seen from Figure 9 and Figure 10 that the internal potential of the device is highly consistent with the charge collection law, so it can be concluded that the position with the largest internal potential of the GaN HEMTs is the position where the device is most sensitive to single-event transient effects.

**Figure 11:**
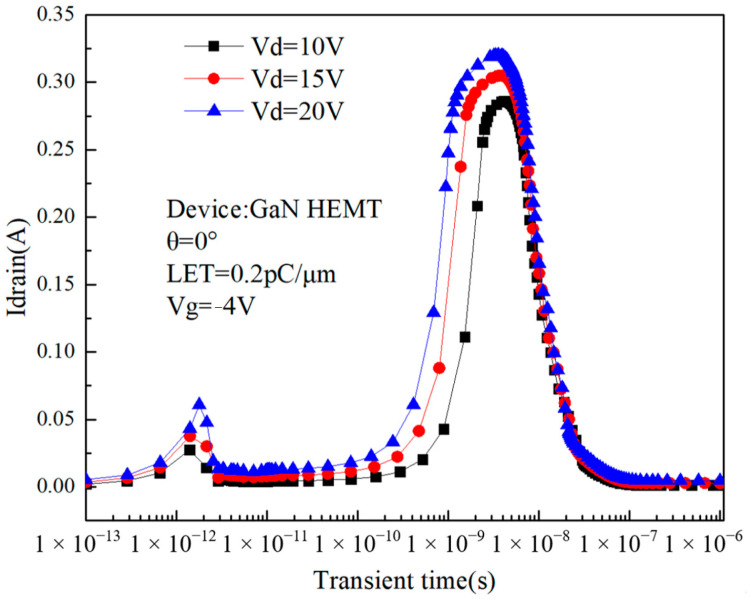
Relationship between drain current and the drain bias voltage over time.

From the analysis above, it can be seen that the internal electric field distribution of the device has a great influence on charge collection. The transient effects of GaN HEMTs are studied by changing the drain bias and then changing the internal electric field of the device. The simulation result is shown in Figure 11.

**Figure 12:**
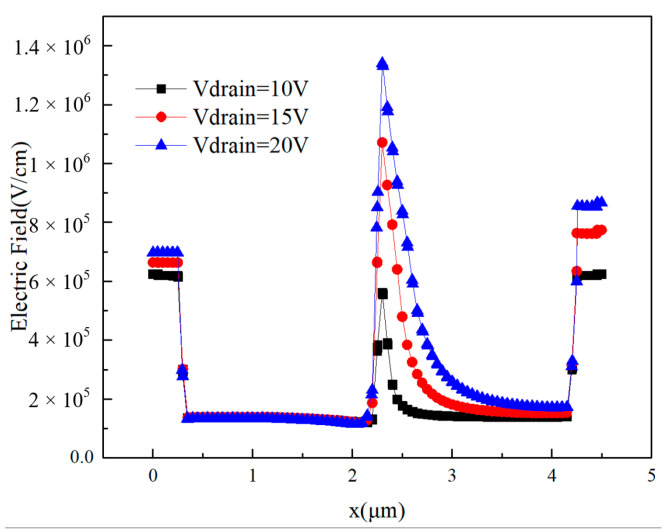
The distribution of electric field inside GaN HEMT along the direction of the 2DEG when the drain bias voltage changes.

As can be seen from Figure 11, as the drain bias voltage increases, so does the peak drain transient current. When the drain voltage is 10V, the first current peak is 0.028 A, and the second current peak is 0.286 A; and when the drain voltage is 20 V, the first current peak is 0.061 A, and the second current peak is 0.320 A. The peak increased by 117% and 12%, respectively. Figure 12 shows the distribution of electric field strength in the device along the direction of the 2DEG channel when the drain voltages are 10 V, 15 V, and 20 V, respectively. It can be seen from the figure that the increase in drain voltage leads to an increase in the electric field strength inside the device, especially near the gate region and the drain region; the increase in the electric field strength makes it easier for the electrode to collect electron–hole pairs, and the increase in the collected charge leads to an increase in the transient peak current. Abbate and Ngom et al. experimentally compared the single-event transient current of GaN HEMTs with different drain voltages [24,25], and the results are consistent with the conclusions of this paper.

Compared with the case where the drain voltage is low, when the drain voltage is high, the drain current increases accordingly. It can be seen from the figure that the increase in the drain voltage leads to an increase in the strength of the electric field near the gate region, as well as an increase in the drift velocity of electrons and holes, making it easier for holes to accumulate near the buffer layer below the gate, further strengthening the backchannel effect and advancing the occurrence time of charge enhancement. This is manifested by an earlier increase in the drain current. Back-channel effect means that when heavy ions incident into the device, it will ionize a large number of electron–hole pairs near its incidence path; when a higher bias voltage is applied to the drain, the device will generate an electric field from the drain to the gate and the source, and this electric field will drive electrons and holes to drift motion because the mobility of electrons is high, meaning they can move to the drain quickly so that they are collected by the drain, generating transient current, and the holes mobility is low, so they can only slowly move towards the gate. As a result, a higher concentration of holes accumulates under the gate, and the positive charge carried by the holes lowers the barrier between the region below the gate and the source, eventually allowing additional electrons to flow from the source into the device and participate in charge collection.

**Figure 13:**
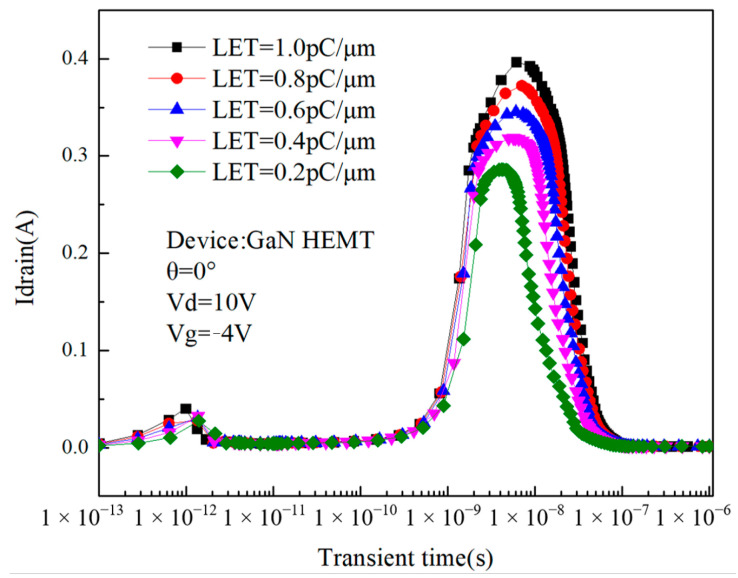
Relationship between drain current and the LET values over time.

The single-event transient effects of the device are not only related to the device state but also related to the LET values of the incident heavy ion (the linear energy transfer capacity of the heavy ion; LET refers to the transfer of energy between particles and the impacted substance due to collisions when ionizing radiation penetrates a substance, which is directly related to the number of electron–hole pairs that the particle can ionize), the angle of incidence, and other conditions. Figure 13 shows the effect of LET values on single-event transient currents. For heavy ions in space, the LET values range is about 0.2~1.0 pC/μm, according to which the relationship between the single-event transient current of GaN HEMT with time is simulated when the LET values is within this range.

It can be seen from the figure that the peak height of the transient current increases with the increase in the LET values of the incident heavy ion. The simulation results are similar to those reached by Rostewitz et al. using GaN HEMT experiments [11]. When the LET value is 0.2 pC/μm, the second peak current is 0.286 A, and when the LET value is 1.0 pC/μm, the second peak current reaches 0.397 A, which is an increase of 38.8%. For a determined material, due to its average ionization energy determination, when the heavy ion is incident, the larger its LET value, the greater the number of electron–hole pairs it can ionize, the more charge collected by the drain, and the higher the peak current. As the LET value increases, the pulse width increases, but the drain current does not increase earlier. This is because the change in LET values does not cause a change in the internal electric field distribution of the device and does not affect the back-channel effect of the device, so the time of charge enhancement does not change.

**Figure 14:**
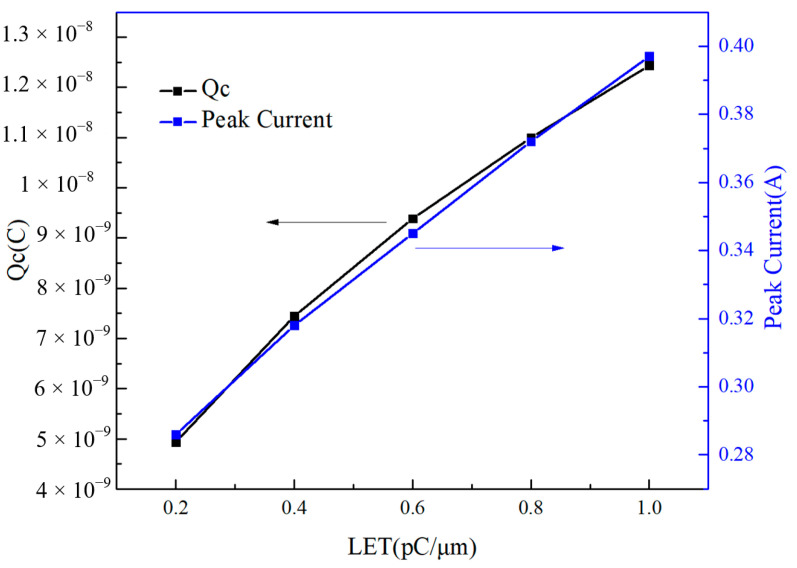
The relationship between the amount of charge collected by the device, the second transient current peak, and the LET values of the incident heavy ion.

The amount of charge collected by the device, the second transient current peak, and the LET values of the incident heavy ion are shown in Figure 14. Since the first peak duration is extremely short and the peak height is small, it is negligible. It can be seen from the figure that the effect of single-event transient effects on GaN HEMTs has a strong correlation with the incident heavy ion LET values.

**Figure 15:**
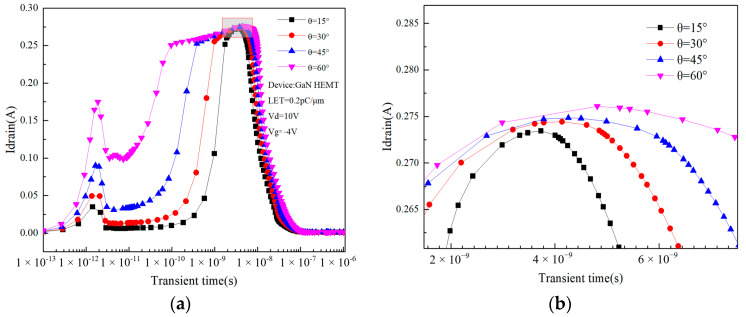
(**a**) Relationship between drain current and the angle of incidence over time. (**b**) Local magnification of peak drain current.

The effect of incident heavy ion angle on GaN HEMT is simulated as shown in Figure 15a,b. It can be seen that as the incidence angle of heavy ion increases, the transient current peak width increases significantly, but the peak change is minimal. When the incidence angle is 15°, the peak drain transient current is the smallest, which is 0.273 A, and when the incidence angle is 60°, the peak drain transient current is the maximum, which is 0.276 A.

**Figure 16:**
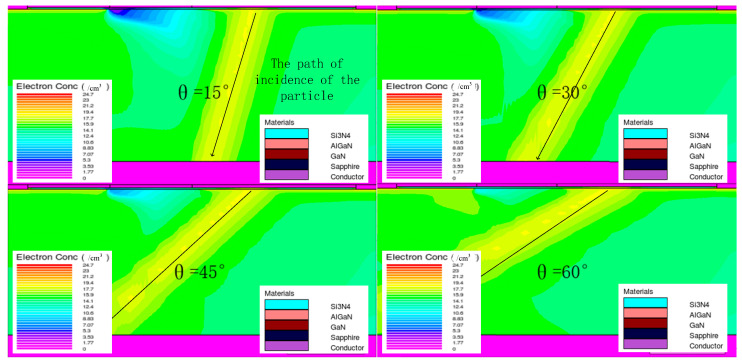
Incidence of heavy ion at different angles.

The change in the amount of charge collected is mainly due to the different incidence paths of the heavy ion, resulting in different numbers of ionized electron–hole pairs. Figure 16 shows the incidence of heavy ion at different angles of incidence. In the model established herein, the heavy ion incidence path is a cylinder, and the cylinder volume can be expressed as:

(5) $$V=\frac{\pi r^{2}h}{\cos^{2}\theta }$$

As the angle of the incident heavy ion increases, the volume of the cylinder formed by the path of the incident heavy ion also increases correspondingly, resulting in an increase in the number of ionized electron–hole pairs on the path, thereby increasing the number of charges collected.

For the first transient current peak, as the angle of incidence of the heavy ion increases, the heavy ion incidence trajectory gets closer and closer to the gate, resulting in an increase in the amount of ionized charge collected by the gate near the incident trajectory, which is manifested as an increase in the first current peak. For the second transient current peak, the current peak width reflects the time it takes for the electron–hole pairs to be collected by the source and drain. As the incidence angle of the heavy ion increases, the ionization path deviates more and more from the sensitive position of the device, resulting in longer charge collection and increased transient current peak duration.

## 4. Conclusions

In this paper, the single-event transient effects of GaN HEMT under different conditions are studied. The simulation results show that the single-event transient effects of the GaN HEMTs in on-state and off-state are different, and the device is more seriously affected when it is in off-state. When the heavy ion is incident, there will be two peaks in the transient current successively; the first peak is due to the electron–hole pairs being collected by the gate and drain, and the later peak is due to the electron–hole pairs being collected by the source and drain. For GaN HEMTs, the sensitive location of single-event transient effects is the region near the drain edge. As the drain voltage increases, the peak values of the single-event transient current increase. Because the field strength increases with the drain bias, the time for charge enhancement to occur is correspondingly earlier. As the LET value of the incident heavy ion increases, so does the drain current pulse width, which is caused by an increase in the number of electron–hole pairs ionized by the incident heavy ion. As the angle of the incident heavy ion increases, the peak variation in the drain current is small; however, the pulse width increases significantly because when the angle of incidence increases, the ionization trajectory is far away from the sensitive area of the device, meaning that the charge collection time increases.

## Figures and Tables

**Table 1:** Device structure parameters during simulation.

Parameters of GaN Material	Values
Thickness of AlGaN barrier/μm	0.02
Thickness of GaN buffer/μm	1.94
Width of gate/μm	50
Length of gate/μm	1

**Table 2:** The parameters of GaN material in simulation.

Parameters of GaN Material	GaN
The band gap energy	3.4/eV
The relative dielectric permittivity	9.5
Electron low-field mobility in GaN layer	900/(cm^2^/(V*s))
Saturated velocity of electrons	2 × 10^7^/(cm/s)

## Data Availability

The data presented in this study are available on request from the corresponding author.
